# Transcription factors in megakaryocytes and platelets

**DOI:** 10.3389/fimmu.2023.1140501

**Published:** 2023-03-09

**Authors:** Hengjie Yuan, Yafan Liu, Jianning Zhang, Jing-fei Dong, Zilong Zhao

**Affiliations:** ^1^Tianjin Institute of Neurology, Department of Neurosurgery, Tianjin Medical University General Hospital, Tianjin, China; ^2^BloodWorks Research Institute, Seattle, WA, United States; ^3^Division of Hematology, Department of Medicine, University of Washington, School of Medicine, Seattle, WA, United States

**Keywords:** transcription factor, platelet reactivity, platelet-microparticles, thrombosis, non-transcriptional manner

## Abstract

Transcription factors bind promoter or regulatory sequences of a gene to regulate its rate of transcription. However, they are also detected in anucleated platelets. The transcription factors RUNX1, GATA1, STAT3, NFκB, and PPAR have been widely reported to play key roles in the pathophysiology of platelet hyper-reactivity, thrombosis, and atherosclerosis. These non-transcriptional activities are independent of gene transcription or protein synthesis but their underlying mechanisms of action remain poorly defined. Genetic and acquired defects in these transcription factors are associated with the production of platelet microvesicles that are known to initiate and propagate coagulation and to promote thrombosis. In this review, we summarize recent developments in the study of transcription factors in platelet generation, reactivity, and production of microvesicles, with a focus on non-transcriptional activities of selected transcription factors.

## 1 Introduction

Transcription factors (TFs) are a group of mediators that bind the promoter or regulatory sequence of a gene to control its rate of transcribing genetic information from DNA to messenger RNA (1). This transcription control is key to ensuring an adequate level of expression of a given protein in targeted cells at a particular developmental stage. It not only directs the processes of proliferation, growth, and death of a cell, but also controls the rate of cell migration and organizational development during embryonic development, as well as regulating cellular response to the extracellular matrices. Thus far, more than 1600 transcription factors have so far been identified (2, 3), and they work in a coordinated fashion to down- as well as up-regulate target genes. The activation of a given gene can be regulated by multiple transcriptional factors and one transcription factor can regulate multiple genes. Such a multivalent activity is possible because of the modular structure of a transcriptional factor, which typically includes a DNA-binding domain, signal-sensing domain that contains binding sites for transcription co-regulators, and an optional transactivation domain, which senses external signals and transmits them to the rest of the transcription complex (4, 5). Because of their roles in regulating gene transcription, the activation and suppression of transcription factors is extensively reported in cancer development (6).

Paradoxically, multiple transcription factors have been reported to express and be active in platelets (**Table 1**), the anucleated offspring of megakaryocytes with a very limited capacity for protein synthesis (7). An obvious question is whether these transcription factors are merely leftover from parental megakaryocytes or have unique activities in platelets. Reports from studies on platelet transcription factors have been scarce in the literature, but increasing evidence suggests that transcription factors in platelets have unique activities of their own independent of their transcriptional activities (8–10). However, past research on transcription factors in platelets is often limited to reporting their presence and activation status, without further investigation of their activities in regulating platelet functions and, more importantly the underlying mechanism of their regulatory activities.

**Table 1:** Roles of transcription factors in platelets.

Transcription factor	Roles under activation or mutation	Associated hematologic abnormalities
RUNX1	Platelet granule development, platelet activation	MDS/AML
GATA1	Inhibit aggregation	Dyserythropoiesis
STAT3	Increase aggregation, P-selectin, thrombosis	Coronary artery diseases
NFkB	Increase aggregation, spreading, clot retraction, GPIBa shedding	Cardiovascular diseases
PPAR	Inhibition of platelet function	Cardiovascular diseases

AML, acute myeloid leukemia; MDS, myelodysplastic syndrome.

Platelets circulate along the vessel wall and act to stop bleeding at sites of vessel injury. This hemostatic process requires multiple ligand-receptor interactions to tether, activate, and aggregate platelets. The tightly controlled platelet activation and aggregation that occurs at the site of vascular injury during hemostasis can become dysregulated in pathological conditions, promoting thrombosis and inflammation. For example, platelets promote arterial thrombosis or thromboembolism when activated either on the surface of a ruptured atherosclerotic plaque or by pathological levels of high fluid shear stress in the area of arterial stenosis, leading to acute thrombotic events such as ischemic stroke and myocardial infarction (11). Emerging evidence further suggests that platelets also act as a cellular mediator in a variety of pathophysiological conditions such as cancer, rheumatoid arthritis, atherosclerosis, trauma, and immune response (12–14). How transcription factors regulate platelet production from megakaryocytes has been extensively reported, but their non-transcriptional activities (i.e., activity independent of gene regulations) have only begun to be recognized. Here, we discuss several transcription factors that have been reported to regulate platelet production and function.

**Figure 1:**
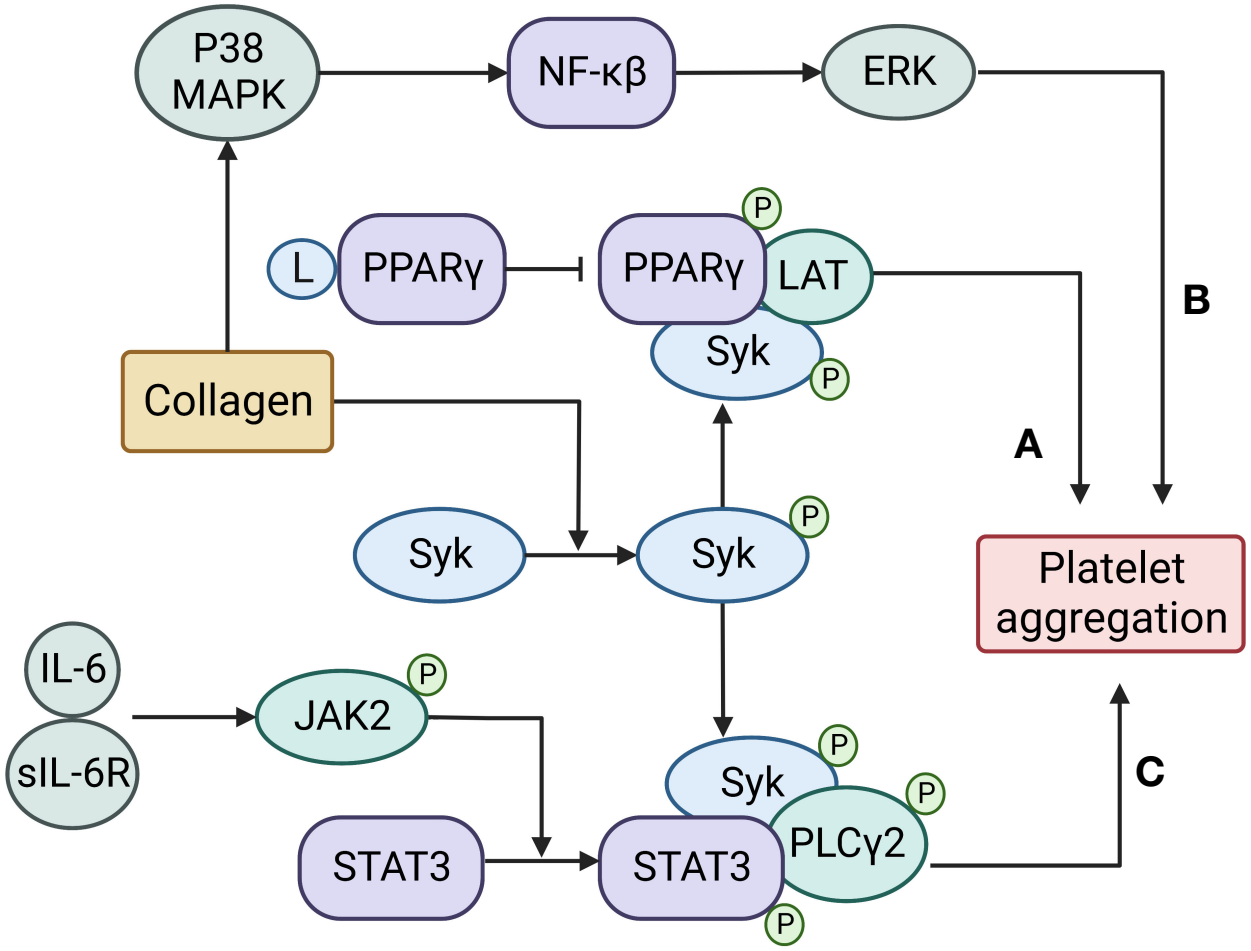
Transcription factors regulate platelet aggregation through non-transcriptional activities. **(A)** PPARγ is recruited and phosphorylated by Syk to promote the recruitment of LAT and enhance platelet aggregation;**(B)** NFκB is activated by upstream p38 mitogen-activated protein kinase (MAPK) and promotes platelet aggregation by regulating downstream extracellular signal-regulated kinase (ERK);**(C)** A complex of IL-6 with its soluble receptor IL-6R activates JAK2 to phosphorylate and dimerize STAT3, then the activated STAT3 serves as a protein scaffold to facilitate the catalytic interaction between the spleen tyrosine kinase (Syk) and its substrate PLCγ2 to promote platelet aggregation.

## 2 Transcription factors in platelet production

### 2.1 Runt-related transcription factor 1

In 1969, Weiss, et al. identified a family with an autosomal dominant inherited thrombocytopenia, caused primarily by decreased dense granule contents (15). A heterozygous Y260X mutation in the RUNX1 gene was subsequently shown to be the genetic basis of this inherited platelet defect (15, 16). To date, more than 200 families with RUNX1 variants have been reported (17). RUNX1/AML1 (also known as CBFA2 and PEBP2αB) is a member of the Runt family, which has three known transcription factors (RUNX1, RUNX2, and RUNX3), which share the Runt homology domain near the N-terminus. This domain interacts with CBFb to bind specific sequences of DNA to regulate its transcription (18).

RUNX1 regulates several genes that control platelet production, structure, function, and intracellular signaling. One report found that 22 patients in a family with autosomal dominant thrombocytopenia had mutations in the RUNX1 gene (19) and 6 of them developed hematologic malignancies (20). RUNX1-deficient mice die in uterus due to defective hematopoiesis and resultant severe bleeding (21, 22). Mice with the conditional knockout survive but have an impaired megakaryocyte maturation with a significant reduction in megakaryocyte polyploidization (23). Variations in the RUNX1 gene often result in bleeding diathesis, primarily because of defective platelet granules (15, 16), which reduce platelet activation and aggregation (24). For example, mice carrying the RUNX1 p.Leu43Ser variant (equivalent to human p.Leu56Ser) exhibit a prolonged bleeding time because of defective α-granule secretion and platelet spreading (25).*RUNX1* deficiency can result in pallidin dysregulation and deficient dense granules in platelets (26) as well as the Ras-related protein RAB31-mediated early endosomal trafficking of von Willebrand factor (VWF) and epidermal growth factor receptor (EGFR) in megakaryocytes (27). RUNX1 regulates the development of platelet granules through interaction with genes involved in the biogenesis of platelet granules such as the nuclear factor erythroid 2 (NF-E2).

In addition, RUNX1 can also regulate genes related to platelet functions. For example, it regulates the transcription of the non-muscle myosin IIA (MYH9) and IIB (MYH10) genes, which encode non-muscle myosin II heavy chains; RUNX1 mutations are associated with dysregulated expression of MYH10 in platelets (28); and the expression level of non-muscle myosin is used as a marker for changes in transcriptional activity of RUNX1 as well as friend leukemia integration 1 transcription factor (FLI1) (29). RUNX1 also regulates the expression of the arachidonate 12-lipoxygenase gene (ALOX12) (30), which encodes the enzyme that acts on polyunsaturated fatty acid substrates to generate bioactive lipid mediators to regulate platelet function (30). PCTP (phosphatidylcholine transfer protein) regulates the intermembrane transfer of phosphatidylcholine and its upregulation by RUNX1 sensitizes platelet response to thrombin through protease-activated receptor 4 (31). RUNX1 also regulates the expression of platelet factor 4 through coordination with transcription factors in the ETS family that share a conserved winged helix-turn-helix DNA binding domain that recognizes unique DNA sequences containing GGAA/T (32). Platelet factor 4 belongs to the CXC chemokine family and is released from α-granules of activated platelets to promote coagulation and to participate in heparin-induced thrombocytopenia (33, 34). A recent report shows that RUNX-1 haploinsufficiency inhibits the differentiation of hematopoietic progenitor cells (HPCs) into megakaryocytes (35).

### 2.2 GATA-binding protein 1

GATA-binding protein 1 (GATA1) is a transcription factor that contains two zinc finger domains: a C-terminal zinc finger that binds the (T/A) GATA(A/G) motif of DNA and an N-terminal zinc finger that is required for stabilizing the C-terminal structure and also interacts with a nuclear co-factor protein called friend for GATA1 (FOG1), which stabilizes GATA1 binding (36, 37). GATA plays a pivotal role in hematopoietic development and is found in megakaryocytes (38). GATA1-deficient mice die before birth at approximately embryonic day 10, primarily because of severe anemia (39). However, mutations in the N-terminal zinc finger domain, which reduces the transcriptional activation of GATA1 (36, 40), are found in patients with myeloproliferative disorders and acute megakaryoblastic leukemia (41), suggesting that GATA1-FOG1 interaction is essential for the development and maturation of megakaryocytes, the parental cells of platelets. Decreased GATA-1 expression has also been reported in patients with myelodysplastic syndrome (42).

Embryonic stem cells from GATA1-deficient mice are smaller and show low expression of megakaryocytic markers, but have a high rate of proliferation (43). Complementation of these cells with a wild-type GATA1 gene allows megakaryocytes and erythrocytes to develop in response to a variety of cytokines. Additionally, cell division is attenuated in the megakaryocytic progenitor G1ME cells that overexpress GATA1. A recent report further shows that impaired MYH10 silencing causes GATA1-related polyploidization defect during megakaryocyte differentiation (44).

Furthermore, platelet aggregation induced by collagen is inhibited in GATA1*-*deficient mice (45), primarily due to reduced expression of the collagen receptor GPVI. Platelet adhesion and aggregation induced by shear stress are also reduced in GATA1*-*deficient mice (45). How a GATA1 deficiency causes these changes in platelet reactivity remains unknown, but these phenotypic changes in the mice provide the first indication that transcription factors could perform non-transcriptional activities in anucleated platelets.

## 3 Non-transcriptional activity in platelets

### 3.1 Signal transducer and activator of transcription 3

STAT includes a family of transcription factors critical for inflammatory and acute-phase reactions (46, 47). They also play vital roles in cancer development and hematopoiesis (48). The homologous STAT1, STAT3, and STAT5 are expressed in human platelets and are reported to regulate platelet reactivity through residual or mitochondrial transcriptional activity in platelets. For example, STAT3 affects mitochondrial transcription by binding to the regulatory D-loop region of mitochondrial DNA upon platelet activation (49).

However, STAT3 can also be activated (phosphorylated) and dimerized in platelets stimulated with thrombopoietin (49, 50), suggesting that STAT3 can also regulate platelet reactivity through non-transcriptional means. We have shown that STAT3 is activated and dimerized in collagen-stimulated platelets to serve as a protein scaffold that facilitates the catalytic interaction between spleen tyrosine kinase (Syk) and its substrate, PLCγ to enhance collagen-induced calcium mobilization and platelet activation (8). More importantly, STAT3 is activated to form dimers by a complex of IL-6 with its soluble receptor IL-6Rα, which activates JAK2 (51). The pharmacological inhibition of platelet STAT3 reduces collagen-induced platelet aggregation and thrombus formation on the collagen matrix (8, 52). Platelets from STAT3-deficient mice or mice infused with a STAT3 inhibitor have reduced collagen-induced aggregation. This non-transcriptional activity of STAT3 may be critical for the development of platelet hyper-reactivity, which has been widely associated with inflammation, especially that related to the activity of the proinflammatory cytokine IL-6 (8). We have also shown that the *piper longum* derivative piperlongumine (PL) blocks collagen-induced platelet reactivity in a dose-dependent manner by targeting STAT3 (53). Consistent with our observations, the small molecular STAT3 inhibitor SC99 has been shown to reduce platelet activation and aggregation induced by collagen and thrombin (54). These findings offer a new pathway for reducing platelet hyper-reactivity in conditions of inflammation and in prothrombotic states associated with trauma, cancer, autoimmune diseases, and severe infection.

### 3.2 Nuclear factor kappa β

Nuclear factor kappa β (NFκB) is a well-defined redox-sensitive transcription factor that regulates the immune response and inflammation by controlling the expression of multiple genes activated by inflammatory mediators (55–57). Blocking NFκB can therefore improve outcomes of inflammatory diseases (58). NFκB is composed of p50 and p65 subunits, normally as an inactive cytoplasmic complex. The inhibitory proteins of the IκB family tightly bind the subunits of NFκB (59). Upon activation, the IκK complex phosphorylates IκBα, thus activating NFκB by detaching it from IkBα (60–62). Three IκK family members, α, β, and γ, are expressed in platelets, with β being the most abundant, and are reported to regulate platelet reactivity through non-transcriptional activity (9, 10, 63). For example, the pharmacological inhibition of IκKβ leads to reduced agonist-induced platelet activation, increased bleeding time, and prolonged thrombus formation in a mouse model (64). NF-κB has also been reported to be partially involved in the regulation of SERCA activity to regulate calcium homeostasis in platelets (65). IκKβ-deficient platelets lose the ability to shed the ectodomain of GP Ibα in response to ADP or collagen stimulations (66) but preserve thrombin-induced GP Ibα shedding (67). Collagen-induced p65 and IκKβ phosphorylation is blocked by inhibition of MAP kinase, but not by inhibition of ERK in platelets (68). The thrombin-induced GP Ibα shedding requires p38 mitogen-activated protein kinase (MAPK) and extracellular signal-regulated kinase (ERK) as its upstream and downstream molecules (68, 69).

### 3.3 Peroxisome proliferator-activated receptors

The peroxisome proliferator-activated receptors (PPARs) are ligand-activated receptors in the nuclear hormone receptor family. They contain three subtypes (PPARα, PPARβ/δ, and PPARγ), which are essential in the regulation of cell differentiation, development, and metabolism (70–72). All PPARs heterodimerize with retinoid X receptor (RXR) and subsequently bind to a specific region of target genes called a peroxisome proliferator response element (PPRE) (73). PPARγ plays a transcription factor role in regulating platelet production from megakaryocytes, but the PPARγ ligand thiazolidinedione inhibits platelet aggregation induced by ADP under hydrostatic pressure and in diabetic mice (74–76). Similarly, activating PPARβ/δ also reduces platelet reactivity to ADP, thrombin, and collagen (77, 78). However, PPARα is also required for platelet activation and thrombus formation, in which it regulates the dense granule secretion of platelets in hyperlipidemic mice (79). The reason for this apparent contradiction remains to be further investigated. PPARγ is recruited and phosphorylated by Syk to promote the recruitment of the protein called Linker for the Activation of T cells (LAT), which is necessary for collagen-induced platelet activation through glycoprotein VI (80).

While transcription factors are critically involved in megakaryocyte development and platelet production, they may also regulate platelet reactivity to conventional and specific platelet agonists (**Figure 1**). The latter is independent of transcriptional activity, for which it is present but at a residual level. This non-transcriptional activity remains poorly understood and requires further investigation because it helps understanding how platelets are activated either by conventional agonists for hemostasis or as complications found in patients treated with drugs that block transcriptional activity of cells (e.g., cancer treatments). Such research will also play an important role in developing new therapeutics targeting these transcription factors to enhance or reduce platelet reactivity.

## 4 Transcription factors in extracellular vesicles released from platelets

Extracellular vesicles (EVs) are shed membrane fragments, intracellular organelles, and nuclear components from cells undergoing active microvesiculation (81–84) or apoptosis (85–87). The former is triggered by the activation of the cysteine protease calpain, which disrupts the membrane-cytoskeleton association (88–91). Platelets are the primary source of EVs circulating in blood, accounting for approximately 80% of total EVs (92–94). The subcellular size of EVs allows them to travel to areas where parental cells are unable to go. In additional to inherent functions from their parental cells, EVs also perform unique activities of their own because of molecules expressed on their surface and carried by them, the latter of which include transcription factors such as STAT3, STAT5, and PPARγ (95) as well as regulators of transcription factors (96, 97). This EV-derived transcriptional activity has been scarcely reported but hold greats potential for influencing biological activities of target cells. For example, PPARγ in platelet EVs is taken up by monocytic THP-1 cells, where it induces the expression of fatty acid-binding protein-4 (FABP4). Monocytes receiving PPARγ-containing platelet EVs produce less inflammatory mediators and become more adherent through increased fibronectin production (95). Although reports on platelet-derived transcription factors remain very limited, a large body of evidence in the literature shows that platelet-derived EVs, especially EV-carried microRNAs, can change transcriptional activities, thus regulating the function of target cells. Platelet EV-carried NLR family pyrin domain containing 3 (NLRP3) stimulates endothelial cells to undergo pyroptosis through the NLRP3/nuclear factor (NF)-κB pathway (98). EVs from platelets stimulated with bacteria provoke proinflammatory activity of monocytes through the TRAF6/NFκB pathway (99). MicroRNA-142-3p carried by platelet-derived EVs promotes the proliferation of endothelial cells (100), whereas microRNA-126-3p-carrying platelet EVs can be internalized by macrophages to dose-dependently downregulate expression of target mRNA (101). These observations mostly pertain to phenotypic characterization with less information regarding the underlying pathways involved. Systemic studies of EV-carrying transcription factors and related mediators are therefore urgently needed.

## 5 Conclusion

Platelets lack a nucleus and *de novo* transcription, but a number of transcription factors are found in platelets and may have non-transcriptional activities that regulate platelet function. Transferring transcription factors between platelets and target cells through platelet EVs could also be a novel regulatory mechanism of cell-cell communications and a potential therapeutic target for a variety of pathologies.

## Author contributions

HY and YL performed the literature search and compiled all the information from the researched articles and wrote the manuscript. ZZ, J-FD and JZ formulated, proposed, guided and wrote the manuscript. All authors contributed to the article and approved the submitted version.
